# Report on the 5‘th scientific meeting of the “Verein zur Förderung des Wissenschaftlichen Nachwuchses in der Neurologie” (NEUROWIND e.V.) held in Motzen, Germany, Oct. 25th – Oct. 27th, 2013

**DOI:** 10.1186/2040-7378-5-15

**Published:** 2013-12-11

**Authors:** Ralf A Linker, Tim Magnus, Thomas Korn, Christoph Kleinschnitz, Sven G Meuth

**Affiliations:** 1 Department of Neurology, Friedrich-Alexander-Universität Erlangen, Schwabachanlage 6, Erlangen 91054, Germany; 2 Department of Neurology, University Clinic Hamburg-Eppendorf, Martinistr. 52, Hamburg 20246, Germany; 3 Department of Neurology, Klinikum rechts der Isar, Technische Universität München, Ismaninger Str. 22, München 81675, Germany; 4 Department of Neurology, University Clinic of Würzburg, Josef-Schneider-Str. 11, Würzburg 97080, Germany; 5 Department of Neurology, University of Münster, Albert-Schweitzer Campus 1, Münster 48149, Germany

## Abstract

From october 25th - 27th 2013, the 5th NEUROWIND e.V. meeting was held in Motzen, Brandenburg, Germany. This year more than 60 doctoral students and postdocs from over 25 different groups working in German university hospitals or research institutes attended the meeting to discuss their latest findings in the fields of neuroimmunology, neurodegeneration and neurovascular research. All participants appreciated the stimulating environment in Motzen, Brandenburg, and people took the opportunity for scientific exchange, discussion about ongoing projects and already started further collaborations. Like in the previous years, the symposium was regarded as a very well organized platform to support research of young investigators in Germany.

According to the major aim of NEUROWIND e.V. to support younger researchers in Germany the 3rd NEUROWIND YOUNG SCIENTIST AWARD for experimental neurology was awarded to Ruth Stassart working in the group of Klaus Armin Nave and Wolfgang Brück (MPI Göttingen and Department of Neuropathology, Göttingen Germany). The successful work was published in *Nature Neuroscience* entitled “*A role for Swann cell*-*derived neuregulin*-*1 in remyelination*”. This outstanding paper deals with the function of Schwann cell neuregulin as an endogenous factor for myelin repair. The award is endowed with 20.000 Euro sponsored by Merck Serono GmbH, Darmstadt, Germany (unrestricted educational grant).

This year’s keynote lecture was given by Albert Ludolph, Head of the Department of Neurology at the University Clinic of Ulm. Dr. Ludolph highlighted the particular role of individual scientists for the development of research concepts in Alzheimer´s disease (AD) and frontotemporal dementia (FTD).

## Summary of the scientific contributions to the NEUROWIND e.V. meeting 2013

### Contributions on vascular neurology

Johanna Ruhnau from Alexander Dressel`s group in Greifswald reported on the effects of cerebral ischemia on monocyte and granulocyte function in cell culture assays from stroke patients. Next, Gilla Lättig from the group of Christoph Harms, Berlin, gave a talk on neuroprotective effects by E2 factor mediated ORC-1 repression in an oxygen glucose deprivation cell culture model. Peter Ludewig from Tim Magnus` group studied the role of carcinoembryonic antigen-related cell adhesion molecule 1 (CEACAM-1) in experimental stroke. Lack of CEACAM-1 increased infarct size, blood brain barrier breakdown as well as edema formation, which was accompanied by impaired neurologic outcome measures in the middle cerebral artery occlusion (MCAO) model via effects on granulocytes and MMP-9. Tian Zhang from Andreas Meisel`s laboratory in Berlin focused on the effects of late CD4^+^ T cell depletion on vascular remodeling and clinical outcome in a murine MCAO stroke model. Robert Brunkhorst (from Christian Förch`s group, Frankfurt) talked about effects of the sphingosin-1 receptor modulator fingolimod in the later phase after experimental cerebral ischemia. Finally, Eva Göb from the group of Christoph Kleinschnitz in Würzburg focused on the role of NADPH oxidases (NOX) in models of focal cerebral ischemia. She reported on stroke outcome in mice conditionally deficient for NOX4 in endothelial or smooth muscle cells thereby extending the already published data on constitutive *Nox4*^−/−^ mice.

### Neuroimmunology and oligodendroglial biology

Felipe Ortega from Mainz gave a talk on Wnt signalling pathways controlling distinct lineage commitment of subependymal zone stem cells giving rise to either oligodendroglial or neuronal cells. Next, Vasileia-Ismini Alexaki from the group of Trian Chavakis, Dresden, reported on the role of the tyrosine kinase receptor TrkA and its ligand nerve growth factor as modulator of microglia-mediated inflammation. Eva Tolosa from Hamburg focused on regulatoy T cells (T_reg_) positive for the cell surface marker CD39. These T_reg_ may modulate the ATP/adenosine axis in autoimmune diseases such as multiple sclerosis (MS). Verena Schultz from the group of Andreas Junker in Göttingen gave a presentation on the role of the immunomodulatory surface molecule integrin associated protein (CD47) during remyelination. They studied the role of CD47 for processes of demyelination *in vivo* (cuprizone model) and cell culture models of myelination. Agnes Kovacs from the group of Ralf Linker, Erlangen, talked about the role of alternative renin angiotensin (Ang) system signalling pathways including Ang1-7 and Mas in experimental autoimmune encephalomyelitis (EAE). Next, Thomas Korn from Munich gave a presentation on the consequences of VLA-4 blockade on the function of different CD4^+^ positive T helper cell subsets in a mouse model of acute viral encephalitis. Kerstin Berer from Harmut Wekerle`s lab in Munich investigated the gut microbiota as new trigger for CNS-specific autoimmunity. Tobias Lanz from Michael Platten`s group in Heidelberg presented data on the key role of protein kinase Cβ pathways for blood brain barrier function in EAE. PKCβ inhibitors like enzastaurin may prove to be promising compounds for the treatment of neuroinflammation. Focusing on CD8^+^ positive T cell antigen recognition, Eva Reuter from the group of Volker Siffrin (Mainz) provided data on *in vivo* imaging of cross recognition between OVA and MOG in CD8 T cells during EAE.

Bettina Trinschek from the Jonuleit laboratory in Mainz talked about the role of IL-6 in the regulation of T_reg_ function in humanized mice and MS patients. The „early“ presence of IL-6 may induce resistance of effector T cells towards T_reg_. At the end of the session, Benjamin Schattling from Manuel Friese`s group in Hamburg presented work on the role of transient receptor potential (TRP) ion channels in neuroinflammation. He focused on transient receptor potential melastatin (TRPM) 4 cation channels in neurodegeneration during EAE and TRPM2 in the MCAO model of cerebral ischemia.

### Neuroscience and neurodegeneration

Petra Hühnchen from the group of Wolfgang Böhmerle, Berlin, reported on the behavioral, electrophysiological and morphometric characterization of animal models of neuropathic pain induced with different chemotherapeutics with a focus on allodynia. Janos Groh from Rudolf Martini´s group in Würzburg gave a talk on neurodegeneration and the role of immune cells in the visual system of mouse models for infantile and juvenile neuronal ceroid lipofuscinosis. Focusing on peripheral innate immunity in Parkinon`s disease (PD), Veselin Grozdanov from Karin Danzer`s group in Ulm reported on cytokine profiles and function of isolated monocytes from PD patients. In a talk on sporadic proteopathies of the brain, Markus Krohn from Jens Pahnke`s group in Magdeburg reported on the role of ABC transporters in Alzheimer`s disease with a special focus on the effect of ABCB1 and ABCC1 regarding clearance mechanisms. Judith Alferink from the Department of Psychiatry in Münster investigated APP/PS1 mice and presented data on the role of CCL17 in neurodegeneration. CCL17 deficiency resulted in beneficial microglia responses and rescued mice from cognitive decline. Finally, Nadine Haase from Dominik Müller`s group in Berlin provided a study on alpha1 adrenergic receptor (α1AR) and β-amyloid. Amyloid β peptides may activate α1AR thus providing a possible link between hypertension and Alzheimer`s disease.

## Competing interests

The authors declare that they have no competing interest.

## Authors’ contributions

RL, TM, TK, CK and SGM wrote the paper. All authors read and approved the final manuscript.

## Authors’ information

**List of speakers at the 5**’**th scientific meeting of NEUROWIND e.V. (in alphabetical order)**

Ismini Alexaki, Division of Vascular Inflammation and Diabetes, Department of Internal Medicine and Institute for Clinical Chemistry and Laboratory Medicine, Medical Theoretical Center, University Clinic Carl-Gustav Carus, TU Dresden, Fetscherstraße 74, 01307 Dresden, Germany.

Kerstin Berer, Max-Planck Institute of Neurobiology, Department of Neuroimmunology, Max-Lebsche-Platz 30, 81377 Munich, Germany

Robert Brunkhorst, Klinikum der Johann Wolfgang Goethe-Universität, Zentrum der Neurologie und Neurochirurgie, Klinik für Neurologie, Theodor-Stern-Kai 7, 60590 Frankfurt/Main, Germany.

Eva Göb, Department of Neurology, University Clinic of Würzburg, Josef-Schneider-Str. 11, 97080 Würzburg, Germany.

Janos Groh, Department of Neurology, Developmental Neurobiology, University of Würzburg, Josef-Schneider-Str. 11, 97080 Würzburg, Germany

Veselin Grozdanov, Universität Ulm, Experimentelle Neurologie, Albert-Einstein-Allee 11, O25 R541, 89081 Ulm, Germany.

Nadine Haase, Max-Delbrück-Centrum für Molekulare Medizin Berlin-Buch, ECRC, Forschungsgebäude der Charité, Lindenberger Weg 80, 13125 Berlin, Germany.

Petra Hühnchen, Charité - Universitätsmedizin Berlin, Klinik für Neurologie, Department of Experimental Neurology, Charitéplatz 1, 10117 Berlin, Germany

Agnes Kovacs, Department of Neurology, Friedrich-Alexander-Universität Erlangen, Schwabachanlage 6, 91054 Erlangen, Germany.

Markus Krohn, University and DZNE Magdeburg, Department of Neurology, Neurodegeneration Research Lab (NRL), Leipziger Str 44, Bldg 64, 39120 Magdeburg, Germany.

Gilla Lättig, Charité - Universitätsmedizin Berlin, Klinik für Neurologie, Department of Experimental Neurology, Charitéplatz 1, 10117 Berlin, Germany.

Tobias Lanz, DKFZ, Klinische Kooperationseinheit Neuroimmunologie und Hirntumorimmunologie (G160), Im Neuenheimer Feld 280, 69120 Heidelberg, Germany.

Albert Ludolph, Universitätsklinik für Neurologie Ulm, Oberer Eselsberg 45, 89081 Ulm, Germany.

Kim Neitzert, University of Münster, Klinik und Poliklinik für Psychiatrie und Psychotherapie, Albert-Schweitzer-Straße 11, 48149 Münster, Germany

Felipe Ortega, Institute of Physiological Chemistry, University Medical Center

Johannes Gutenberg University Mainz, Hanns-Dieter-Hüsch-Weg 19, 55128 Mainz, Germany.

Björn Rissiek, Department of Immunology, University Medical Center Hamburg-Eppendorf, Campus Forschung N27, Room 2.055, Martinistrasse 52, 20246 Hamburg, Germany.

Veit Rothhammer, Department of Neurology, Klinikum rechts der Isar, Technische Universität München, Ismaninger Str. 22, 81675 München, Germany.

Johanna Ruhnau, Klinik und Poliklinik für Neurologie, AG Neuroimmunologie, Ferdinand Sauerbruchstraße, 17475 Greifswald, Germany.

Benjamin Schattling, Forschergruppe Neuroimmunologie, Zentrum für Molekulare Neurobiologie, Universitätsklinikum Hamburg-Eppendorf, Falkenried 94, 20251 Hamburg, Germany.

Verena Schultz, University Medical Center Göttingen, Department of Neuropathology, Robert-Koch-Str.40, 37075 Göttingen, Germany.

Eva Tolosa, Department of Immunology, University Medical Center Hamburg-Eppendorf, Campus Forschung N27, Room 2.055, Martinistrasse 52, 20246 Hamburg, Germany.

Bettina Trinschek, Universitätsmedizin Mainz, Hautklinik, Langenbeckstr. 1, 55131 Mainz, Germany.

Tian Zhang, Charité - Universitätsmedizin Berlin, Klinik für Neurologie, Department of Experimental Neurology, Charitéplatz 1, 10117 Berlin, Germany.

Eva Zindler, Klinik für Neurologie, Universitätsmedizin Mainz, Langenbeckstr. 1, 55131 Mainz, Germany.

